# Tweet sentiment quantification: An experimental re-evaluation

**DOI:** 10.1371/journal.pone.0263449

**Published:** 2022-09-16

**Authors:** Alejandro Moreo, Fabrizio Sebastiani

**Affiliations:** Istituto di Scienza e Tecnologie dell’Informazione, Consiglio Nazionale delle Ricerche, Pisa, Italy; European Commission, ITALY

## Abstract

Sentiment quantification is the task of training, by means of supervised learning, estimators of the relative frequency (also called “prevalence”) of sentiment-related classes (such as Positive, Neutral, Negative) in a sample of unlabelled texts. This task is especially important when these texts are tweets, since the final goal of most sentiment classification efforts carried out on Twitter data is actually quantification (and not the classification of individual tweets). It is well-known that solving quantification by means of “classify and count” (i.e., by classifying all unlabelled items by means of a standard classifier and counting the items that have been assigned to a given class) is less than optimal in terms of accuracy, and that more accurate quantification methods exist. Gao and Sebastiani 2016 carried out a systematic comparison of quantification methods on the task of tweet sentiment quantification. In hindsight, we observe that the experimentation carried out in that work was weak, and that the reliability of the conclusions that were drawn from the results is thus questionable. We here re-evaluate those quantification methods (plus a few more modern ones) on exactly the same datasets, this time following a now consolidated and robust experimental protocol (which also involves simulating the presence, in the test data, of class prevalence values very different from those of the training set). This experimental protocol (even without counting the newly added methods) involves a number of experiments 5,775 times larger than that of the original study. Due to the above-mentioned presence, in the test data, of samples characterised by class prevalence values very different from those of the training set, the results of our experiments are dramatically different from those obtained by Gao and Sebastiani, and provide a different, much more solid understanding of the relative strengths and weaknesses of different sentiment quantification methods.

## 1 Introduction

*Quantification* (also known as *supervised prevalence estimation*, or *learning to quantify*) is the task of training (by means of supervised learning) a predictor that estimates the relative frequency (also known as *prevalence*, or *prior probability*) of each class of interest in a set (here often called a “sample”) of unlabelled data items, where the data used to train the predictor are a set of labelled data items [1]. (Throughout the paper we prefer the term “unlabelled text” to the term “test text” because the former embraces not only the case in which we are testing a quantification method in lab experiments, but also the case in which, maybe after performing these experiments, we deploy our trained models in an operational environment in order to perform quantification on the data that our application requires us to analyse.) Quantification finds applications in fields (such as the social sciences [2], epidemiology [3], market research [4], and ecological modelling [5]) that inherently deal with aggregate (rather than individual) data, but is also relevant to other applications such as resource allocation [6], word sense disambiguation [7], and improving classifier fairness [8].

In the realm of textual data, one important domain to which quantification is applied is *sentiment analysis* [9, 10]. In fact, as argued by Esuli et al. [11], many applications of sentiment classification are such that the final goal is not determining the class label (e.g., Positive, or Neutral, or Negative) of an individual unlabelled text (for example, a blog post, a response to an open question, or a comment on a product), but is that of determining the relative frequencies of the classes of interest in a set of unlabelled texts. In a 2016 paper, Gao and Sebastiani [12] (hereafter: [GS2016]) have argued that, when the objects of analysis are tweets, the *vast majority* of sentiment classification efforts actually have quantification as their final goal, since hardly anyone who engages in sentiment classification of tweets is interested *per se* in the sentiment conveyed by a specific tweet. We call the resulting task *tweet sentiment quantification* [11, 11, 13].

It is well-known (see e.g., [1, 6, 14–21]) that solving quantification by means of “classify and count” (i.e., by classifying all the unlabelled items by means of a standard classifier and counting the items that have been assigned to a given class) is less than optimal in terms of quantification accuracy, and that more accurate quantification methods exist. Driven by these considerations, [GS2016] presented an experimental comparison of 8 important quantification methods on 11 Twitter datasets annotated by sentiment, with the goal of assessing the strengths and weaknesses of the various methods for tweet sentiment quantification. That paper became then influential (at the time of writing, [GS2016] and paper [22], a shorter and earlier version of [GS2016], have 134 citations altogether on Google Scholar) and a standard reference on this problem, and describes what is currently the largest comparative experimentation on tweet sentiment quantification.

In this paper we argue that the conclusions drawn from the experimental results obtained in [GS2016] are unreliable, as a result of the fact that the experimentation performed in that paper was weak. We thus present new experiments in which we re-test all 8 quantification methods originally tested in [GS2016] (plus some additional ones that have been proposed since then) on the same 11 datasets used in [GS2016], using a now consolidated and robust experimental protocol. These new experiments (conducted on a set of samples that is at the same time (a) 5,775 times larger than the set of samples used in [GS2016], even without counting the experiments on new quantification methods that had not been considered in [GS2016], and more varied than it) return results dramatically different from those obtained in [GS2016], and give us a new, more reliable picture of the relative merits of the various methods on the tweet sentiment quantification task.

The rest of this paper is structured as follows. In Section 2 we discuss experimental protocols for quantification, and argue why the experimentation carried out in [GS2016] is, in hindsight, weak. In Section 3 we present the new experiments we have run, briefly discussing the quantification methods and the datasets we use, and explaining in detail the experimental protocol we use. Section 4 discusses the results and the conclusions that they allow drawing, also pointing at how they differ from the ones of [GS2016], and why. Section 5 is devoted to concluding remarks.

We make all the code we use for our experiments available (see https://github.com/HLT-ISTI/QuaPy/tree/tweetsent). Together with the fact that [GS2016] made available (in vector form) all their 11 datasets (see https://zenodo.org/record/4255764), this allows our experiments to be easily reproduced by other researchers.

## 2 Experimental protocols for quantification

### 2.1 Notation

In this paper we use the following notation. By **x** we indicate a document drawn from a domain X of documents, while by *y* we indicate a class drawn from a set of classes (also known as a *codeframe*) $Y=\{y_{1},...,y_{\left|Y\right|}\}$. Given x∈X and y∈Y, a pair (**x**, *y*) denotes a document with its true class label. Symbol *σ* denotes a *sample*, i.e., a non-empty set of (labelled or unlabelled) documents drawn from X. By *p*_*σ*_(*y*) we indicate the true prevalence of class *y* in sample *σ*, by ${\hat{p}}_{\sigma }\left(y\right)$ we indicate an estimate of this prevalence, and by ${\hat{p}}_{\sigma }^{M}\left(y\right)$ we indicate the estimate of this prevalence obtained by means of quantification method *M* (consistently with most mathematical literature, we use the caret symbol (^) to indicate estimation). Since 0 ≤ *p*_*σ*_(*y*) ≤ 1 and $0\leq {\hat{p}}_{\sigma }\left(y\right)\leq 1$ for all y∈Y, and since $\sum_{y\in Y}p_{\sigma }\left(y\right)=\sum_{y\in Y}{\hat{p}}_{\sigma }\left(y\right)=1$, the *p*_*σ*_(*y*)’s and the ${\hat{p}}_{\sigma }\left(y\right)$’s form two probability distributions across the same codeframe.

By $D(p,\hat{p})$ we denote an evaluation measure for quantification; these measures are typically *divergences*, i.e., functions that measure the amount of discrepancy between two probability distributions. By *L* we denote a set of labelled documents, that we typically use as a training set, while by *U* we denote a set of unlabelled documents, that we typically use for testing purposes. We take a *hard classifier* to be a function h:X→Y, and a *soft classifier* to be a function $s:X\to {\left[0,1\right]}^{\left|Y\right|}$, where *s*(**x**) is a vector of |Y| *posterior probabilities* (each indicated as Pr(*y*|**x**)), such that $\sum_{y\in Y}\text{Pr}\left(y|x\right)=1$; Pr(*y*|**x**) indicates the probability of membership in *y* of item **x** as estimated by the soft classifier *s*. By *δ*_*σ*_(*y*) we denote the set of documents in sample *σ* that have been assigned to class *y* by a hard classifier.

### 2.2 Why do we need quantification?

Quantification may be seen as the task of training, via supervised learning, a predictor that estimates an unknown *true distribution* *p*_*σ*_, where *p*_*σ*_ is defined on a sample *σ* and across the classes in a codeframe $Y=\{y_{1},...,y_{\left|Y\right|}\}$, by means of a *predicted distribution* ${\hat{p}}_{\sigma }$. In other words, in quantification one needs to generate estimates ${\hat{p}}_{\sigma }\left(y_{1}\right),...,{\hat{p}}_{\sigma }\left(y_{\left|Y\right|}\right)$ of the true (and unknown) class prevalence values $p_{\sigma }\left(y_{1}\right),...,p_{\sigma }\left(y_{\left|Y\right|}\right)$, where $\sum_{y\in Y}{\hat{p}}_{\sigma }\left(y\right)=\sum_{y\in Y}p_{\sigma }\left(y\right)=1$. In this paper we consider a ternary sentiment quantification task (an example of *single-label multiclass quantification*) in which the codeframe is Y={Positive,Neutral,Negative}, and where these three class labels will be indicated, for brevity, by the symbols {⊕, ⊙, ⊖}, respectively. All the 11 datasets discussed in Section 3.5 use this codeframe.

The reason why true quantification methods (i.e., different from the trivial “classify and count” mentioned in Section 1) are needed is the fact that many applicative scenarios suffer from *distribution shift*, the phenomenon according to which the distribution *p*_*L*_(*y*) in the training set *L* may substantially differ from the distribution *p*_*σ*_(*y*) in the sample *σ* of unlabelled documents that one needs to label [23, 24]. The presence of distribution shift means that the well-known IID assumption, on which most learning algorithms for training classifiers are based, does not hold; in turn, this means that “classify and count” will perform less than optimally on samples of unlabelled items that exhibit distribution shift with respect to this training set, and that the higher the amount of shift, the worse we can expect “classify and count” to perform.

### 2.3 The APP and the NPP

There are two main experimental protocols that have been used in the literature for evaluating quantification; we will here call them the *artificial-prevalence protocol* (APP) and the *natural-prevalence protocol* (NPP).

The APP consists of taking a standard dataset (by which we here mean any dataset that has originally been assembled for testing classification systems; any such dataset can be used for testing quantification systems too), split into a training set *L* of labelled items and a set *U* of unlabelled items, and conducting repeated experiments in which either the training set prevalence values of the classes, or the test set prevalence values of the classes, are artificially varied by means of subsampling (i.e., by removing random elements of specific classes until the desired class prevalence values are obtained). In other words, subsampling is used either to generate *s* training samples $\sigma_{1}^{L}\subseteq L,...,\sigma_{s}^{L}\subseteq L$, or to generate *t* test samples $\sigma_{1}^{U}\subseteq U,...,\sigma_{t}^{U}\subseteq U$, or both, where the class prevalence values of the generated samples are predetermined and set in such a way as to generate a wide array of distribution drift values. This is meant to test the robustness of a *quantifier* (i.e., of an estimator of class prevalence values) in scenarios characterized by class prevalence values very different from the ones the quantifier has been trained on. For instance, in the binary quantification experiments carried out in [15], given codeframe $Y=\{y_{1},y_{2}\}$, repeated experiments are conducted in which examples of either *y*_1_ or *y*_2_ are removed at random from the test set in order to generate predetermined prevalence values for *y*_1_ and *y*_2_ in the samples $\sigma_{1}^{U},...,\sigma_{t}^{U}$ thus obtained. In this way, the different samples are characterised by a different prevalence of *y*_1_ (e.g., $p_{\sigma_{i}^{U}}\left(y_{1}\right)\in \left\{0.00,0.05,...,0.95,1.00\right\}$) and, as a result, by a different prevalence of *y*_2_. This can be repeated, thus generating multiple random samples for each chosen pair of class prevalence values. Analogously, random removal of examples of either *y*_1_ or *y*_2_ can be performed on the training set, thus bringing about training samples with different values of $p_{\sigma_{i}^{L}}\left(y_{1}\right)$ and $p_{\sigma_{i}^{L}}\left(y_{2}\right)$.

This protocol has been criticised (see [25]) because it may generate samples exhibiting class prevalence values very different from the ones of the set (*L* or *U*) from which the sample *σ* was extracted, i.e., class prevalence values that might be hardly plausible in practice. As a result, one may resort to the NPP, which consists instead of doing away with sample extraction and directly using, as the samples for conducting the experiments, the test set *U* (or portions of it obtained by subdividing it into bins) and the training set *L* that have been sampled IID from the data distribution. In other words, no perturbation of the original class prevalence values is performed for extracting samples. An example experimentation that uses the NPP is the one reported in [25], where the authors test binary quantifiers on 52 × 99 = 5,148 samples. This results from the fact that, in using the RCV1-v2 test collection, they consider the 99 RCV1-v2 classes and bin the 791,607 test documents in 52 bins (each corresponding to a week’s worth of data, since the RCV1-v2 data span one year) of 15,212 documents each on average, and use the resulting bins as the samples. However, it is not always easy to find test collections with such a large amount of classes and annotated data, and this limits the applicability of the NPP. (It should also be mentioned that, as Card and Smith [26] noted, the vast majority of the 5,148 RCV1-v2 *test samples* used in [25] exhibit very little distribution shift, which makes the testbed used in [25] unchallenging for quantification methods).

The experimentation conducted by [GS2016] on tweet sentiment quantification is also an example of the NPP, since it relies on 11 datasets of tweets annotated by sentiment from which no extraction of samples at prespecified values of class prevalence was performed. For each dataset, the authors use the training set *L* as the sample *σ*_*L*_ on which to train the quantifiers, and the test set *U* as the sample *σ*_*U*_ on which to test them. However, what the authors of [GS2016] overlooked is that, while in classification an experiment involving 11 different datasets probably counts as large and robust, this does not hold in quantification *if only one test sample per dataset is used*. The reason is that, since the objects of quantification are *sets* (i.e., samples) of documents in the same way that the objects of classification are individual documents, testing a tweet sentiment quantifier on just 11 samples should be considered, from an experimental point of view, a drastically insufficient experimentation, akin to testing a tweet sentiment classifier on 11 tweets only.

As a result, we should conclude that the authors of [GS2016] (unintentionally) carried out a weak evaluation, and that the results of that experimentation are thus unreliable. We thus re-evaluate the same quantification methods that [GS2016] tested (plus some other more recent ones) on the same datasets, this time following the robust and by now consolidated APP; in our case, this turns out to involve 5,775 as many experiments as run in the original study, even without considering the experiments on quantification methods that had not been considered in [GS2016]).

It might be argued that the APP is unrealistic because it generates samples whose class prevalence values are too far away from the values seen in the set from where they have been extracted, and that such scenarios are thus unlikely to occur in real applicative settings. However, in the absence of any prior knowledge about how the class prevalence values are allowed or expected to change in future data, the APP turns out to be not only the fairest protocol, since it relies on no assumptions that could penalize or benefit any particular method, but also the most interesting for quantification, since quantification is especially useful in cases of distribution shift.

Yet another way of saying this comes from the observation that, should we adopt the NPP instead of the APP, a method that trivially returns, as the class prevalence estimates for *every* test sample, the class prevalence values from the training set (this trivial method is commonly known in the quantification literature as the *maximum likelihood prevalence estimator* – MLPE), would probably perform well, and might even beat all genuinely engineered quantification methods. The reason why it would probably perform well is that the expectations of the class prevalence values of samples drawn IID from the test set coincide with the class prevalence values of the test set, and these, again by virtue of the IID assumption, are likely to be close to those of the training set. In other words, the reason why MLPE typically performs well when evaluated according to the NPP, does not lie in the (inexistent) qualities of MLPE as a quantification method, but in the fact that the NPP is a weak evaluation protocol.

## 3 Experiments

In this section we describe the experiments we have carried out in order to re-assess the merits of different quantification methods under the lens of the APP. We have conducted all these experiments using QuaPy (see https://github.com/HLT-ISTI/QuaPy), a software framework for quantification written in Python that we have developed and made available through GitHub (see branch tweetsent).

### 3.1 Evaluation measures

As the measures of quantification error we use *Absolute Error* (AE) and *Relative Absolute Error* (RAE), defined as

$$\begin{array}{c} \text{AE}(p,\hat{p})=\frac{1}{\left|Y\right|}\sum_{y\in Y}\left|\hat{p}\left(y\right)-p\left(y\right)\right| \end{array}$$ (1)

$$\begin{array}{c} \text{RAE}(p,\hat{p})=\frac{1}{\left|Y\right|}\sum_{y\in Y}\frac{|\hat{p}\left(y\right)-p\left(y\right)|}{p(y)} \end{array}$$ (2)

where *p* is the true distribution, $\hat{p}$ is the estimated distribution, and Y is the set of classes of interest (Y={⊕,⊙,⊖} in our case). (The sample *σ* on which we quantify is left implicit in order not to overload the notation).

Note that RAE is undefined when at least one of the classes y∈Y is such that its prevalence in the sample *σ* is 0. To solve this problem, in computing RAE we smooth all *p*(*y*)’s and $\hat{p}\left(y\right)$’s by means of additive smoothing, i.e., we compute

$$\begin{array}{c} \underline{p}\left(y\right)=\frac{\epsilon +p(y)}{\epsilon |Y|+\sum_{y\epsilon Y}p\left(y\right)} \end{array}$$ (3)

where $\underline{p}\left(y\right)$ denotes the smoothed version of *p*(*y*) and the denominator is just a normalising factor (same for the $\hat{\underline{p}}\left(y\right)$’s); following [6], we use the quantity *ϵ* = 1/(2|*σ*|) as the smoothing factor. We then use the smoothed versions of *p*(*y*) and $\hat{p}\left(y\right)$ in place of their original non-smoothed versions in Eq 2; as a result, RAE is now always defined.

The reason why we use AE and RAE is that from a theoretical standpoint they are, as it has been recently argued [27], the most satisfactory evaluation measures for quantification. This means that we do not consider other measures used in [GS2016], such as KLD, NAE, NRAE, and NKLD, since [27] shows them to be inadequate for evaluating quantification.

### 3.2 Quantification methods used in [GS2016]

We now briefly describe the quantification methods used in [GS2016], that we also use in this paper.

The simplest quantification method (and the one that acts as a lower-bound baseline for all quantification methods) is the above-mentioned *Classify and Count* (**CC**), which, given a hard classifier *h*, consists of computing

$$\begin{array}{c} \begin{array}{cc} {\hat{p}}_{\sigma }^{\text{CC}}\left(y_{i}\right) & =\frac{\left|\{x\in \sigma |h\left(x\right)=y_{i}\}\right|}{\left|\sigma \right|}=\frac{\sum_{y_{j}\in Y}C_{ij}^{h}}{\left|\sigma \right|} \end{array} \end{array}$$ (4)

where $C_{ij}^{h}$ indicates the number of documents classified as *y*_*i*_ by *h* and whose true label is *y*_*j*_. CC is an example of an *aggregative* quantification method, i.e., a method that requires the (hard or soft) classification of all the unlabelled items as an intermediate step. All the methods discussed in this section are aggregative.

The *Adjusted Classify and Count* (**ACC**) quantification method (see [6, 28]) derives from the observation that, by the law of total probability, it holds that

$$\begin{array}{c} \text{Pr}\left(\delta \left(y_{i}\right)\right)=\sum_{y_{j}\in Y}\text{Pr}\left(\delta \left(y_{i}\right)|y_{j}\right)\cdot \text{Pr}\left(y_{j}\right) \end{array}$$ (5)

where *δ*(*y*_*i*_) denotes (see Section 2.1) the set of documents that have been assigned to class *y*_*i*_ by the hard classifier *h*. Eq 5 can be more conveniently rewritten as

$$\begin{array}{c} \frac{\sum_{y_{j}\in Y}C_{ij}^{h}}{\left|\sigma \right|}=\sum_{y_{j}\in Y}\frac{C_{ij}^{h}}{\sum_{y_{x}\in Y}C_{xj}^{h}}\cdot p_{\sigma }\left(y_{j}\right) \end{array}$$ (6)

Note that the leftmost factor of Eq 6 is known (it is the fraction of documents that the classifier has assigned to class *y*_*i*_, i.e., ${\hat{p}}_{\sigma }^{\text{CC}}\left(y_{i}\right)$), and that $C_{ij}^{h}/\sum_{y_{x}\in Y}C_{xj}^{h}$ (which represents the disposition of the classifier to assign *y*_*i*_ when *y*_*j*_ is the true label), while unknown, can be estimated by *k*-fold cross-validation on *L*. Note also that *p*_*σ*_(*y*_*j*_) is unknown (it is the goal of quantification to estimate it), and that there are |Y| instances of Eq 5, one for each $y_{i}\in Y$. We are then in the presence of a system of |Y| linear equations in |Y| unknowns (the *p*_*σ*_(*y*_*j*_)’s); ACC thus consists of estimating these latter (i.e., computing ${\hat{p}}_{\sigma }^{\text{ACC}}\left(y_{j}\right)$) by solving, by means of the known techniques, this system of linear equations.

CC and ACC use the predictions generated by the hard classifier *h*, as evident by the fact that both Eqs 4 and 6 depend on $C_{ij}^{h}$. Since most classifiers can be configured to return “soft predictions” in the form of posterior probabilities Pr(*y*|**x**) (from which hard predictions are obtained by choosing the *y* for which Pr(*y*|**x**) is maximised), and since posterior probabilities contain richer information than hard predictions, it makes sense to try and generate probabilistic versions of the CC and ACC methods [29] by replacing “hard” counts $C_{ij}^{h}$ with their expected values, i.e., with $C_{ij}^{s}=\sum_{(x,y_{j})\in \sigma }\text{Pr}\left(y_{i}|x\right)$. If a classifier natively outputs classification scores that are not probabilities, the former can be converted into the latter by means of “probability calibration”; see e.g., [30].

One can thus define *Probabilistic Classify and Count* (**PCC**) as

$$\begin{array}{c} \begin{array}{cc} {\hat{p}}_{\sigma }^{\text{PCC}}\left(y_{i}\right) & =\frac{\sum_{x\in \sigma }\text{Pr}\left(y_{i}|x\right)}{\left|\sigma \right|}=\frac{\sum_{y_{j}\in Y}C_{ij}^{s}}{\left|\sigma \right|} \end{array} \end{array}$$ (7)

and *Probabilistic Adjusted Classify and Count* (**PACC**), which consists of estimating *p*_*σ*_(*y*_*j*_) (i.e., computing ${\hat{p}}_{\sigma }^{\text{PACC}}\left(y_{j}\right)$) by solving the system of |Y| linear equations in |Y| unknowns

$$\begin{array}{c} \frac{\sum_{y_{j}\in Y}C_{ij}^{s}}{\left|\sigma \right|}=\sum_{y_{j}\in Y}\frac{C_{ij}^{s}}{\sum_{y_{x}\in Y}C_{xj}^{s}}\cdot p_{\sigma }\left(y_{j}\right) \end{array}$$ (8)

The fact that PCC is a probabilistic version of CC is evident from the structural similarity between Eqs 4 and 7, which only differ for the fact that the hard classifier *h* of Eq 4 is replaced by a soft classifier *s* in Eq 7; the same goes for ACC and PACC, as evident from the structural similarity of Eqs 6 and 8.

A further method that [GS2016] uses is the one proposed in [31] (which we here call **SLD**, from the names of its proposers, and which was called EMQ in [GS2016]), which consists of training a probabilistic classifier and then using the EM algorithm (i) to update (in an iterative, mutually recursive way) the posterior probabilities that the classifier returns, and (ii) to re-estimate the class prevalence values of the test set, until mutual consistency, defined as the situation in which

$$\begin{array}{c} p_{\sigma }\left(y\right)\approx \sum_{x\in \sigma }\text{Pr}\left(y|x\right) \end{array}$$ (9)

is achieved for all y∈Y.

Quantification methods **SVM(KLD)**, **SVM(NKLD)**, **SVM(Q)**, belong instead to the “structured output learning” camp. Each of them is the result of instantiating the SVM_perf_ structured output learner [32] to optimise a different loss function. SVM(KLD) [25] minimises the Kullback-Leibler Divergence (KLD); SVM(NKLD) [33] minimises a version of KLD normalised by means of the logistic function; SVM(Q) [34] minimises Q, the harmonic mean of a classification-oriented loss (recall) and a quantification-oriented loss (RAE). Each of these learners generates a “quantification-oriented” classifier, and the quantification method consists of performing CC by using this classifier. These three learners inherently generate *binary* quantifiers (since SVM_perf_ is an algorithm for learning binary predictors only), but we adapt them to work on single-label multiclass quantification. This adaptation consists of training one binary quantifier for each class in Y={⊕,⊙,⊖} by applying a one-vs-all strategy. Once applied to a sample, these three binary quantifiers produce a vector of three estimated prevalence values, one for each class in Y={⊕,⊙,⊖}; we then L1-normalize this vector so as to make the three class prevalence estimates sum up to one (this is also the strategy followed in [GS2016]).

### 3.3 Additional quantification methods

From the “structured output learning” camp we also consider **SVM(AE)** and **SVM(RAE)**, i.e., variants of the above-mentioned methods that minimise (instead of KLD, NKLD, or Q) the AE and RAE measures, since these latter are, for reasons discussed in Section 3.1, the evaluation measures used in this paper for evaluating the quantification accuracy of our systems. We consider SVM(AE) only when using AE as the evaluation measure, and we consider SVM(RAE) only when using RAE as the evaluation measure; this obeys the principle that a sensible user, after deciding the evaluation measure to use for their experiments, would instantiate SVM_perf_ with that measure, and not with others. (Quantification is a task in which deciding the right evaluation measure to use for one’s application is of critical importance; in fact, [27] argues that some applications demand measures such as AE, while the requirements of other applications are best mirrored in measures such as RAE.) These methods have never been used before in the literature, but are obvious variants of the last three methods we have described.

We also include two methods based on the notion of *quantification ensemble* [18, 35]. Each such ensemble consists of *n* base quantifiers, trained from randomly drawn samples of *q* documents each, where these samples are characterised by different class prevalence values. At testing time, class prevalence values are estimated as the average of the estimates returned by the base members of the ensemble. We include two ensemble-based methods recently proposed by Pérez-Gállego et al. [35]; in both methods, a selection of members for inclusion in the final ensemble is performed before computing the final estimate. The first method we consider is **E(PACC)**_Ptr_, a method based on an ensemble of PACC-based quantifiers to which a dynamic selection policy is applied. This policy consists of selecting the *n*/2 base quantifiers that have been trained on the *n*/2 samples characterised by the prevalence values most similar to the one being tested upon (where similarity was previously estimated using all members in the ensemble). We further consider **E(PACC)**_AE_, a method which performs a static selection of the *n*/2 members that deliver the smallest absolute error on the training samples. In our experiments we use *n*=50 and *q*=1,000.

We also report results for **HDy** [36], a probabilistic binary quantification method that views quantification as the problem of minimising the divergence (measured in terms of the Hellinger Distance) between two cumulative distributions of posterior probabilities returned by the classifier, one coming from the unlabelled examples and the other coming from a validation set. HDy looks for the mixture parameter *α* that best fits the validation distribution (consisting of a mixture of a “positive” and a “negative” distribution) to the unlabelled distribution, and returns *α* as the estimated prevalence of the positive class. We adapt the model to the single-label multiclass scenario by using the one-vs-all strategy as described above for the methods based on SVM_perf_.

ACC and PACC define two simple linear *adjustments* to be applied to the aggregated scores returned by general-purpose classifiers. We also use a more recently proposed adjustment method based on deep learning, called **QuaNet** [37]. QuaNet models a neural *non-linear* adjustment by taking as input (i) all the class prevalence values as estimated by CC, ACC, PCC, PACC, and SLD; (ii) the posterior probabilities Pr(*y*|**x**) for each document **x** and for each class y∈Y, and (iii) embedded representations of the documents. As the method for generating the document embeddings we simply perform principal component analysis and retain the 100 most informative components. (Note that, since the datasets we use are available not in raw form but in vector form, we cannot resort to common methods for generating document embeddings, e.g., methods that use recurrent, convolutional, or transformer architectures that directly process the raw text.) QuaNet relies on a recurrent neural network (a bidirectional LSTM) to produce “sample embeddings” (i.e., dense, multi-dimensional representations of the test samples as observed from the input data), which are then concatenated with the class prevalence estimates obtained by CC, ACC, PCC, PACC, and SLD, and then used to generate the final prevalence estimates by transforming this vector through a set of feed-forward layers (of size 1,024 and 512), followed by ReLU activations and dropout (with drop probability set to 0.5).

### 3.4 Underlying classifiers

Consistently with [GS2016], as the classifier underlying CC, ACC, PCC, PACC, and SLD, we use one trained by means of L2-regularised logistic regression (LR); we also do the same for E(PACC)_Ptr_, E(PACC)_AE_, HDy, and QuaNet. The reasons of this choice are the same as described in [GS2016], i.e., the fact that logistic regression is known to deliver very good classification accuracy across a variety of application domains, and the fact that a classifier trained by means of LR returns posterior probabilities that tend to be fairly well-calibrated, a fact which is of fundamental importance for methods such as PCC, PACC, SLD, HDy, and QuaNet. By using the same learner used in [GS2016] we also allow a more direct comparison of results.

As specified above, the classifier underlying SVM(KLD), SVM(NKLD), SVM(Q), SVM(AE), SVM(RAE), is one trained by means of SVM_perf_.

### 3.5 Datasets

The datasets on which we run our experiments are the same 11 datasets on which the experiments of [GS2016] were carried out, and whose characteristics are described succinctly in Table 1. As already noted at the end of Section 1, [GS2016] makes these datasets available already in vector form; we refer to [GS2016] for a fuller description of these datasets.

**Table 1:** Datasets used in this work and their main characteristics. Columns *L*_Tr_, *L*_Va_, *U* contain the numbers of tweets in the training set, held-out validation set, and test set, respectively. Column “Shift” contains the values of distribution shift between *L* ≡ *L*_Tr_ ⋃ *L*_Va_ and *U*, measured in terms of absolute error, columns *p*_*L*_(⊕), *p*_*L*_(⊙), and *p*_*L*_(⊖) contain the class prevalence values of our three classes of interest in the training set *L*, while columns *p*_*U*_(⊕), *p*_*U*_(⊙), and *p*_*U*_(⊖) contain the class prevalence values for the unlabelled set *U*.

Dataset	*L*_Tr_	*L*_Va_	*U*	Total	Shift	*p*_*L*_(⊕)	*p*_*L*_(⊙)	*p*_*L*_(⊖)	*p*_*U*_(⊕)	*p*_*U*_(⊙)	*p*_*U*_(⊖)
GASP	7,532	1,256	3,765	12,553	0.0094	0.421	0.496	0.082	0.407	0.507	0.086
HCR	797	797	798	2,392	0.0630	0.546	0.211	0.243	0.640	0.167	0.193
OMD	1,576	263	787	2,626	0.0171	0.463	0.271	0.266	0.437	0.283	0.280
Sanders	1,847	308	923	3,078	0.0020	0.161	0.691	0.148	0.164	0.688	0.148
SemEval2013	9,684	1,654	3,813	15,151	0.0270	0.159	0.470	0.372	0.158	0.430	0.412
SemEval2014	9,684	1,654	1,853	13,191	0.1055	0.159	0.470	0.372	0.109	0.361	0.530
SemEval2015	9,684	1,654	2,390	13,728	0.0417	0.159	0.470	0.372	0.153	0.413	0.434
SemEval2016	6,000	2,000	2,000	10,000	0.0070	0.157	0.351	0.492	0.163	0.341	0.497
SST	2,546	425	1,271	4,242	0.0357	0.261	0.452	0.288	0.207	0.481	0.312
WA	1,872	312	936	3,120	0.0208	0.305	0.414	0.281	0.282	0.446	0.272
WB	3,650	609	1,823	6,082	0.0023	0.270	0.392	0.337	0.274	0.392	0.335
Average	4,988	994	1,851	7,833	0.0301	0.278	0.426	0.296	0.272	0.410	0.318

Note that [GS2016] had generated these vectors by using state-of-the-art, tweet-specific preprocessing, which included, e.g., URL normalisation, detection of exclamation and/or question marks, emoticon recognition, and computation of “the number of all-caps tokens, (…), the number of hashtags, the number of negated contexts, the number of sequences of exclamation and/or question marks, and the number of elongated words” [GS2016, §4.1]; in other words, every effort was made in [GS2016] to squeeze every little bit of information from these tweets, in a tweet-specific way, in order to enhance accuracy as much as possible.

In the experiments described in this paper we perform feature selection by discarding all features that occur in fewer than 5 training documents.

According to the principles of the APP, as described in Section 2.3, for each of the 11 datasets we here extract multiple samples from the test set, according to the following protocol. For each different triple (*p*(⊕), *p*(⊙), *p*(⊖)) of class prevalence values in the finite set *P* = {0.00, 0.05, …, 0.95, 1.00} and such that the three values sum up to 1, we extract *m* random samples of *q* documents each such that the extracted samples exhibit the class prevalence values described by the triple. In these experiments we use *m* = 25 and *q* = 100. For each label *y* ∈ {⊕, ⊙, ⊖} and for each sample, the extraction is carried out by means of sampling without replacement. (Here it is possible to always use sampling without replacement because each test set contains at least *q* = 100 documents for each label *y* ∈ {⊕, ⊙, ⊖}. If a certain test set contained fewer than *q* = 100 documents for some label *y* ∈ {⊕, ⊙, ⊖}, for that label and that test set it would be necessary to use sampling with replacement.)

It is easy to verify that there exist |*P*|(|*P*| + 1)/2 = 231 different triples with values in *P*. (This follows from the fact that, when *p*_*σ*_(⊕) = 0.00, there exist 21 different pairs (*p*_*σ*_(⊙), *p*_*σ*_(⊖)) with values in *P*; when *p*_*σ*_(⊕) = 0.05, there exist 20 different such pairs; …; and when *p*_*σ*_(⊕) = 1.00, there exists just 1 such pair. The total number of combinations is thus $\sum_{i=1}^{21}i=\frac{21\cdot 22}{2}=231$.) Our experimentation of a given quantification method *M* on a given dataset thus consists of training *M* on the training tweets *L*_Tr_, using the validation tweets *L*_Va_ for optimising the hyperparameters, retraining *M* on the entire labelled set *L* ≡ *L*_Tr_ ⋃ *L*_Va_ using the optimal hyperparameter values, and testing the trained system on each of the 25 × 231 = 5,775 samples extracted from the test set *U*. This is sharply different from [GS2016], where the experimentation of a quantification method *M* on a given dataset consists of testing the trained system on one sample only, i.e., on the entire set *U*.

### 3.6 Parameter optimisation

Parameter optimisation is an important factor, that could bias, if not carried out properly, a comparative experimentation of different quantification methods. As we have argued elsewhere [38], when the quantification method is of the aggregative type, for this experimentation to be unbiased, not only it is important to optimise the hyperparameters of the classifier that underlies the quantification method, but it is also important that this optimisation is carried out using a quantification-oriented loss, and not a classification-oriented loss.

In order to optimise a quantification-oriented loss it is necessary to test each hyperparameter setting on multiple samples extracted from the held-out validation set, in the style of the evaluation described in Section 3.5. In order to do this, for each combination of class prevalence values we extract, from the held-out validation set of each dataset, *m* samples of *q* documents each, again using class prevalence values in *P* = {0.00, 0.05, …, 0.95, 1.00}. Here we use *m* = 5 and *q* = 100; we use a value of *m* five times smaller than in the evaluation phase (see Section 3.5) in order to keep the computational cost of the parameter optimisation phase within acceptable bounds.

For each label *y* ∈ {⊕, ⊙, ⊖} and for each sample, the extraction is carried out by sampling without replacement if the test set contains at least *p*_*y*_⋅*q* examples, and by sampling with replacement otherwise. (Unlike when extracting samples in the evaluation phase—see Section 3.5, it is here sometimes necessary to use sampling with replacement because, in some dataset, the validation set does not contain at least 100 documents per class).

In the experiments that we report in this paper, the hyperparameter that we optimise is the *C* hyperparameter (that determines the trade-off between the margin and the training error) of both LR and SVM_perf_; for this we carry out a grid search in the range *C* ∈ {10^*i*^}, with *i* ∈ [−4, −3, …, + 4, + 5]. We optimise this parameter by using, as a loss function, either the AE measure (the corresponding results are reported in Table 2) or the RAE measure (Table 3). We evaluate the former batch of experiments only in terms of AE and the latter batch only in terms of RAE, following the principle that, once a user knew the measure to be used in the evaluation, they would carry out the parameter optimisation phase in terms of exactly that measure.

Hereafter, with the notation *M*^*D*^ we will indicate quantification method *M* with the parameters of the learner optimised using measure *D*.

**Table 2:** Values of AE obtained in our experiments; each value is the average across 5,775 values, each obtained on a different sample.

	Methods tested in [GS2016]								Newly added methods				
	CC^AE^	ACC^AE^	PCC^AE^	PACC^AE^	SLD^AE^	SVM(Q)^AE^	SVM(KLD)^AE^	SVM(NKLD)^AE^	SVM(AE)^AE^	$\text{E}{\left(\text{PACC}\right)}_{\text{Ptr}}^{\text{AE}}$	$\text{E}{\left(\text{PACC}\right)}_{\text{AE}}^{\text{AE}}$	HDy^AE^	QuaNet^AE^
GASP	0.093	0.052	0.124	0.044	**0.043**	0.119	0.114	0.110	0.136	0.065	0.049	0.086	0.046
HCR	0.130	0.102	0.158	0.074	0.078	0.150	0.143	0.138	0.158	0.084	0.071^†^	**0.070**	0.099
OMD	0.114	0.086	0.126	0.067	**0.055**	0.141	0.124	0.139	0.116	0.084	0.075	0.119	0.087
Sanders	0.114	0.058	0.138	0.049	**0.045**	0.140	0.141	0.110	0.157	0.076	0.058	0.087	0.079
SemEval13	0.115	0.086	0.143	**0.078**	0.097	0.129	0.144	0.134	0.143	0.102	0.093	0.114	0.078^‡^
SemEval14	0.105	0.060	0.136	**0.054**	0.076	0.127	0.128	0.122	0.134	0.096	0.067	0.083	0.059
SemEval15	0.128	0.103	0.148	0.101^†^	0.104	0.143	0.150	0.145	0.144	0.114	0.112	0.105	**0.098**
SemEval16	0.146	0.147	0.171	0.118	**0.102**	0.167	0.154	0.165	0.178	0.131	0.132	0.167	0.103^‡^
SST	0.110	0.083	0.140	0.057	**0.054**	0.136	0.113	0.128	0.126	0.063	0.054^‡^	0.097	0.069
WA	0.082	0.056	0.082	0.043	**0.037**	0.111	0.100	0.063	0.071	0.043	0.041	0.043	0.053
WB	0.077	0.043	0.083	0.035	**0.032**	0.106	0.084	0.103	0.069	0.048	0.041	0.044	0.046
Average	0.110	0.080^‡^	0.132	**0.065**	0.066^‡^	0.134	0.127	0.123	0.130	0.082^‡^	0.072^‡^	0.092^†^	0.074^‡^

**Boldface** indicates the best method for a given dataset. Superscripts † and ‡ denote the methods (if any) whose scores are *not* statistically significantly different from the best one according to a paired sample, two-tailed t-test at different confidence levels: symbol † indicates that 0.001 < *p*-value <0.05 while symbol ‡ indicates that 0.05 ≤ *p*-value. The absence of any such symbol indicates that *p*-value ≤0.001 (i.e., that the performance of the method is statistically significantly different from that of the best method). For ease of readability, for each dataset we colour-code cells in intense green for the best result, intense red for the worst result, and an interpolated tone for the scores in-between.

**Table 3:** Values of RAE obtained in our experiments; each value is the average across 5,775 values, each obtained on a different sample.

	Methods tested in [GS2016]								Newly added methods				
	CC^RAE^	ACC^RAE^	PCC^RAE^	PACC^RAE^	SLD^RAE^	SVM(Q)^RAE^	SVM(KLD)^RAE^	SVM(NKLD)^RAE^	SVM(RAE)^RAE^	$\text{E}{\left(\text{PACC}\right)}_{\text{RAE}}^{\text{Ptr}}$	$\text{E}{\left(\text{PACC}\right)}_{\text{RAE}}^{\text{RAE}}$	HDy^RAE^	QuaNet^RAE^
GASP	2.850	0.512	3.490	0.722	**0.337**	3.835	3.260	3.461	3.411	2.361	1.402	0.644	4.270
HCR	3.982	1.942	4.151	1.332	**0.454**	4.939	4.236	4.197	4.041	2.169	1.990	0.517	4.214
OMD	3.495	0.884	3.776	0.552	**0.469**	4.578	3.844	4.481	3.295	2.479	1.840	0.881	2.296
Sanders	3.296	0.791	3.687	0.990	**0.432**	4.377	3.596	3.533	3.767	2.342	1.559	0.504	1.943
SemEval13	3.117	1.469	3.720	1.244	**0.491**	3.998	3.743	3.960	3.588	2.162	1.602	1.027	1.712
SemEval14	3.079	1.414	3.699	1.271	**0.325**	4.018	3.535	3.741	3.364	2.261	1.691	0.523	1.384
SemEval15	3.608	1.695	4.022	1.884	**0.889**	4.417	4.031	4.264	3.847	2.552	2.233	1.275	1.866
SemEval16	4.594	2.994	5.191	2.815	**1.216**	5.430	4.880	5.278	4.988	4.090	4.057	1.773	4.026
SST	4.207	0.972	4.226	1.042	0.534^†^	4.446	3.621	3.535	3.804	1.880	1.343	**0.487**	1.783
WA	2.493	0.540	2.706	0.512	**0.313**	3.503	2.814	1.431	1.739	0.948	0.825	0.587	1.280
WB	2.419	0.693	2.560	0.669	**0.233**	3.440	2.525	2.243	1.926	0.975	0.790	0.283	1.205
Average	3.376	1.264	3.748	1.185	**0.518**	4.271	3.644	3.648	3.434	2.202	1.757	0.773^‡^	2.362

**Boldface** indicates the best method for a given dataset. Superscripts † and ‡ denote the methods (if any) whose scores are *not* statistically significantly different from the best one according to a paired sample, two-tailed t-test at different confidence levels: symbol † indicates that 0.001 < *p*-value <0.05 while symbol ‡ indicates that 0.05 ≤ *p*-value. The absence of any such symbol indicates that *p*-value ≤0.001 (i.e., that the performance of the method is statistically significantly different from that of the best method). For ease of readability, for each dataset we colour-code cells in intense green for the best result, intense red for the worst result, and an interpolated tone for the scores in-between.

## 4 Results

Table 2 reports AE results obtained by the quantification methods of Sections 3.2 and 3.3 as tested on the 11 datasets of Section 3.5, while Table 3 does the same for RAE. The tables also report the results of a paired sample, two-tailed t-test that we have run, at different confidence levels, in order to check if other methods are different or not, in a statistically significant sense, from the best-performing one.

An important aspect that emerges from these tables is that the behaviour of the different quantifiers is fairly consistent across our 11 datasets; in other words, when a method is a good performer on one dataset, it tends to be a good performer *on all datasets*. Together with the fact that we test on a large set of samples, and that these are characterised by values of distribution shift across the entire range of all possible such shifts, this allows us to be fairly confident in the conclusions that we draw from these results.

A second observation is that three methods (ACC, PACC, and SLD) stand out, since they perform consistently well across all datasets and for both evaluation measures. In particular, SLD is the best method for 7 out of 11 datasets when testing with AE, and for all 11 datasets when testing with RAE. PACC also performs very well, and is the best performer for 3 out of 11 datasets when testing with AE. The fact that both ACC and PACC tend to perform well shows that the intuition according to which CC predictions should be “adjusted” by estimating the disposition of the classifier to assign class *y*_*i*_ when class *y*_*j*_ is the true label, is valuable and robust to varying levels of distribution shift. The same goes for SLD, although SLD “adjusts” the CC predictions differently, i.e., by enforcing the mutual consistency (described by Eq 9) between the posterior probabilities and the class prevalence estimates.

By contrast, these results show a generally disappointing performance on the part of all methods based on structured output learning, i.e., on the SVM_perf_ learner. Note that the fact that SVM(KLD), SVM(NKLD), SVM(Q) optimise a performance measure different from the one used in the evaluation (AE or RAE) cannot be the cause of this suboptimal performance, since this latter also characterises SVM(AE) when tested with AE as the evaluation measure, and SVM(RAE) when tested with RAE.

CC and PCC do no perform well either. If this was somehow to be expected for CC, this is surprising for PCC, which always performs worse than CC in our experiments, on all datasets and for both performance measures. It would be tempting to conjecture that this might be due to a supposedly insufficient quality of the posterior probabilities returned by the underlying classifier; however, this conjecture is implausible, since the quality of the posterior probabilities did not prevent SLD from displaying sterling performance, and PACC from performing very well.

Contrary to the observations reported in [35], the E(PACC)_Ptr_ and E(PACC)_AE_ ensemble methods fail to improve over the base quantifier (PACC) upon which they are built. The likely reason for this discrepancy is that, while Pérez-Gállego et al. [35] trained the base quantifiers on training samples of the same size as the original training set (i.e., they use *q* = |*L*|), we use smaller training samples (i.e., we use *q* = 1,000) in order to keep training times within reasonable bounds (this is also due to the fact that the datasets we consider in this study are much larger than those used in [35], not only in terms of the number of instances but especially in terms of the number of features). (For instance, our datasets always have a number of features in the tens or hundreds of thousands, while in their case this number if between 3 and 256.)

We now turn to comparing the results of our experiments with the ones reported in [GS2016]. For doing this, for each dataset we rank, in terms of their performance, the 8 quantification methods used in both batches of experiments, and compare the rank positions obtained by each method in the two batches. We only perform a qualitative comparison (i.e., comparing ranks) and not a quantitative one (i.e., comparing the obtained scores) because we think that this latter would be misleading. The reason is that the evaluation carried out in the [GS2016] paper and the one carried out here were run on different data. For example, on dataset GASP and using AE as the evaluation measure, SVM(KLD) obtains 0.017 in [GS2016] and 0.114 in this paper, but these results are not comparable, since the above figures are (i) the result of testing on just 1 sample (the unlabelled set) in [GS2016], and (ii) the result of averaging across the results obtained on the 5,775 samples (extracted from the unlabelled set) described in Section 3.5 in this paper. In general, for the same dataset and evaluation measure, the results reported in this paper are far worse than the ones reported in [GS2016], because the experimental protocol adopted in this paper is far more challenging than the one used in [GS2016] since it involves testing on samples whose distribution is very different from the distribution of the training set.

The results of this comparison are reported in Table 4 (for AE) and Table 5 (for RAE).

**Table 4:** Rank positions of the quantification methods in our AE experiments, and (between parentheses) the rank positions obtained by the same methods in the evaluation of [GS2016].

	Methods tested in [GS2016]							
	CC^AE^	ACC^AE^	PCC^AE^	PACC^AE^	SLD^AE^	SVM(Q)^AE^	SVM(KLD)^AE^	SVM(NKLD)^AE^
GASP	4 (5)	3 (3)	8 (2)	2 (1)	1 (6)	7 (8)	6 (4)	5 (7)
HCR	4 (5)	3 (2)	8 (1)	1 (3)	2 (7)	7 (8)	6 (4)	5 (6)
OMD	4 (6)	3 (3)	6 (1)	2 (2)	1 (8)	8 (7)	5 (4)	7 (5)
Sanders	5 (5)	3 (4)	6 (2)	2 (3)	1 (6)	7 (8)	8 (1)	4 (7)
SemEval13	4 (7)	2 (5)	7 (1)	1 (6)	3 (8)	5 (4)	8 (3)	6 (2)
SemEval14	4 (8)	2 (2)	8 (6)	1 (1)	3 (3)	6 (7)	7 (5)	5 (4)
SemEval15	4 (4)	2 (3)	7 (1)	1 (2)	3 (7)	5 (8)	8 (6)	6 (5)
SemEval16	3 (3)	4 (4)	8 (1)	2 (7)	1 (5)	7 (8)	5 (2)	6 (6)
SST	4 (2)	3 (5)	8 (1)	2 (8)	1 (3)	7 (6)	5 (4)	6 (7)
WA	5 (6)	3 (5)	6 (2)	2 (1)	1 (3)	8 (8)	7 (7)	4 (4)
WB	4 (2)	3 (4)	5 (1)	2 (3)	1 (5)	8 (6)	6 (8)	7 (7)
Average	4.1 (4.8)	2.8 (3.6)	7.0 (1.7)	**1.6** (3.4)	**1.6** (5.5)	6.8 (7.1)	6.5 (4.4)	5.5 (5.5)

**Boldface** indicates the best method in terms of average rank in our APP-based experiments, while underline is used to indicate the same for the NPP-based experiments of [GS2016].

**Table 5:** Rank positions of the quantification methods in our RAE experiments, and (between parentheses) the rank positions obtained by the same methods in the evaluation of [GS2016].

	Methods tested in [GS2016]							
	CC^RAE^	ACC^RAE^	PCC^RAE^	PACC^RAE^	SLD^RAE^	SVM(Q)^RAE^	SVM(KLD)^RAE^	SVM(NKLD)^RAE^
GASP	4 (5)	2 (4)	7 (3)	3 (2)	1 (6)	8 (8)	5 (1)	6 (7)
HCR	4 (4)	3 (2)	5 (1)	2 (3)	1 (7)	8 (8)	7 (5)	6 (6)
OMD	4 (6)	3 (3)	5 (1)	2 (2)	1 (8)	8 (7)	6 (4)	7 (5)
Sanders	4 (5)	2 (4)	7 (1)	3 (3)	1 (6)	8 (8)	6 (2)	5 (7)
SemEval13	4 (7)	3 (3)	5 (1)	2 (4)	1 (8)	8 (6)	6 (2)	7 (5)
SemEval14	4 (4)	3 (2)	6 (8)	2 (3)	1 (6)	8 (7)	5 (1)	7 (5)
SemEval15	4 (3)	2 (5)	5 (1)	3 (2)	1 (4)	8 (8)	6 (6)	7 (7)
SemEval16	4 (3)	3 (5)	6 (2)	2 (7)	1 (4)	8 (8)	5 (1)	7 (6)
SST	6 (3)	2 (5)	7 (1)	3 (6)	1 (2)	8 (7)	5 (4)	4 (8)
WA	5 (5)	3 (4)	6 (2)	2 (1)	1 (3)	8 (8)	7 (7)	4 (6)
WB	5 (2)	3 (4)	7 (1)	2 (3)	1 (5)	8 (6)	6 (8)	4 (7)
Average	4.4 (4.3)	2.6 (3.7)	6.0 (2.0)	2.4 (3.3)	**1.0** (5.4)	8.0 (7.4)	5.8 (3.7)	5.8 (6.3)

**Boldface** indicates the best method in terms of average rank in our APP-based experiments, while underline is used to indicate the same for the NPP-based experiments of [GS2016].

Something that jumps to the eye when observing these tables is that our experiments lead to conclusions that are *dramatically different* from those drawn by [GS2016]. First, SLD now unquestionably emerges as the best performer, while it was often ranked among the worst performers in [GS2016]. Conversely, PCC was the winner on most combinations (dataset, measure) in [GS2016], while our experiments have shown it to be a bad performer. Other methods too see their merits disconfirmed by our experiments; in particular, ACC and PACC have climbed up the ranked list, while all other methods (especially SVM(KLD)) have lost ground.

The reason for the different conclusions that these two batches of experiments allow drawing is, in all evidence, the amounts of distribution shift which the methods have had to confront in the two scenarios. In the experiments of [GS2016] this shift was very moderate, since the only test sample used (which coincided with the entire test set) usually displayed class prevalence values not too different from the class prevalence values in the training set. This is shown in the last column of Table 1, where the shift between training set and test set (expressed in terms of absolute error) is reported for each dataset; shift values range between 0.0020 and 0.1055, with an average value across all datasets of 0.0301, which is a very low value. In our experiments, instead, the quantification methods need to confront class prevalence values that are sometimes *very* different from the ones in the training set; shift values range between 0.0000 and 0.6666, with an average value across all samples of 0.2350. This means that the quantification methods that have emerged in our experiments are the ones that are robust to possibly radical changes in these class prevalence values, while the ones that had fared well in the experiments of [GS2016] are the methods that tend to perform well merely in scenarios where these changes are bland.

**Fig 1:**
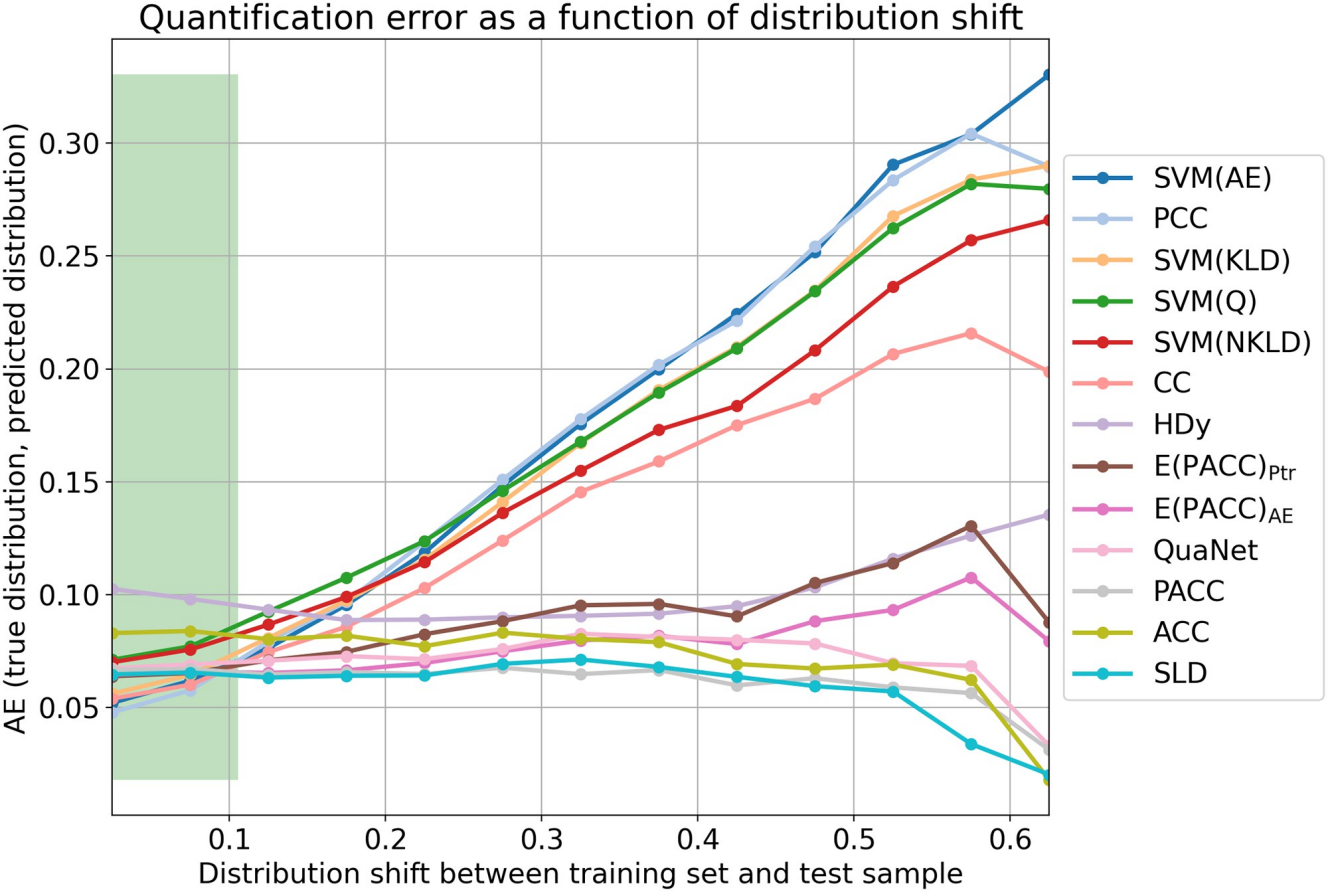
Performance of the various quantification methods, represented by the coloured lines and measured in terms of AE (lower is better), as a function of the distribution shift between training set and test sample; the results are averages across all samples in the same bin, i.e., characterised by approximately the same amount of shift, independently of the dataset they were sampled from. The two vertical dotted lines indicate the range of distribution shift values exhibited by the experiments of [GS2016] (i.e., in those experiments, the AE values of distribution shift range between 0.020 and 0.1055). The green histogram in the background shows instead how the samples we have tested upon are distributed across the different bins.

**Fig 2:**
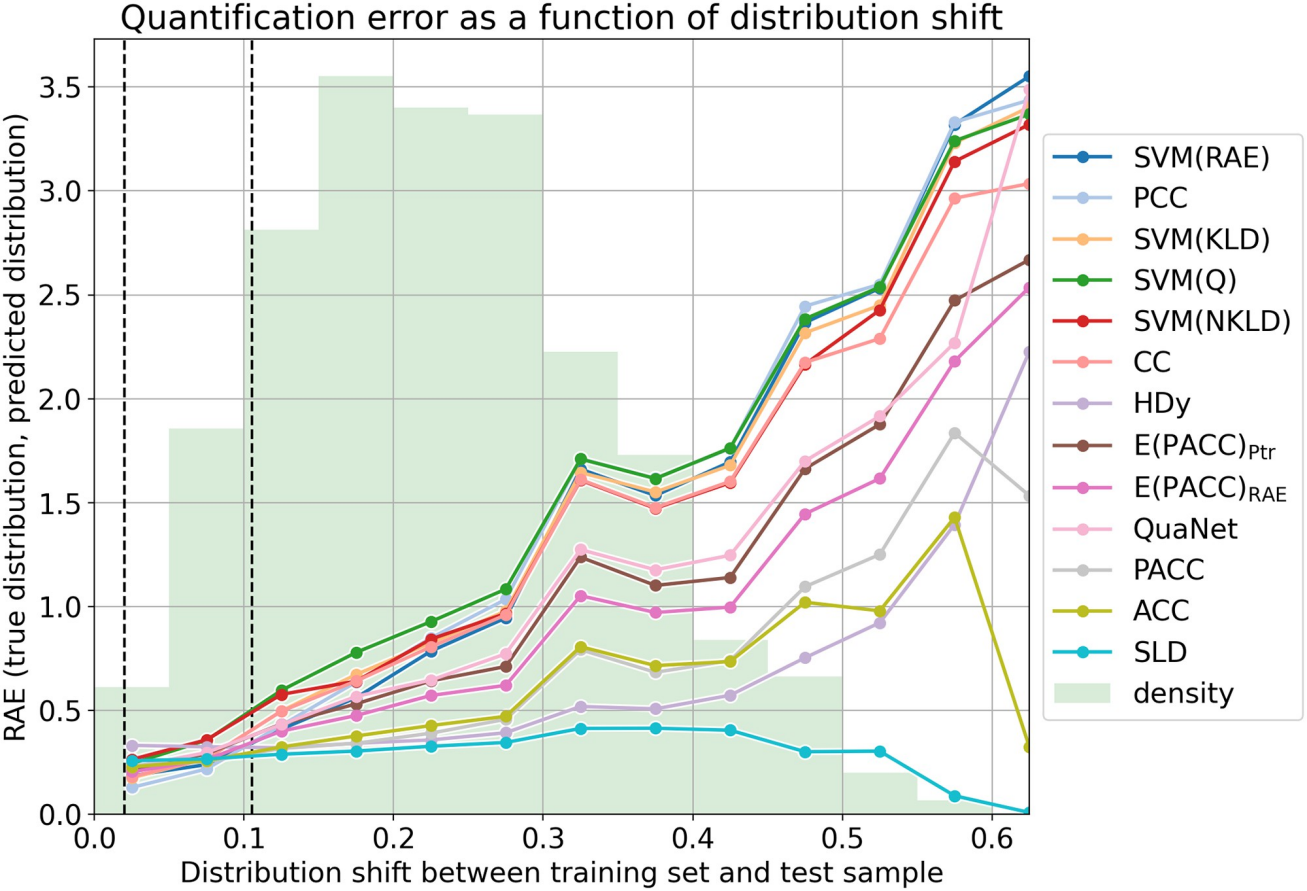
Performance of the various quantification methods, represented by the coloured lines and measured in terms of RAE (lower is better), as a function of the distribution shift between training set and test sample; the results are averages across all samples in the same bin, i.e., characterised by approximately the same amount of shift, independently of the dataset they were sampled from. Unlike in Fig 1, for better clarity these results are actually displayed on a logarithmic scale. The two vertical dotted lines indicate the range of distribution shift values exhibited by the experiments of [GS2016] (i.e., in those experiments, the AE values of distribution shift range between 0.020 and 0.1055). The green histogram in the background shows instead how the samples we have tested upon are distributed across the different bins.

This situation is well depicted in the plots of Figs 1 and 2. For generating these plots we have computed, for each of the 11 × 5,775 = 63,525 test samples, the distribution shift between the training set and the test sample, and we have binned these 63,525 samples into bins characterised by approximately the same amount of distribution shift (we compute distribution shift as the absolute error between the training distribution and the distribution of the test sample, using bins of width equal to 0.05 (i.e., [0.00,0.05], (0.05,0.10], etc.). The plots show, for a given quantification method and for a given bin, the quantification error of the method, measured (by means of AE in the top figure and by means of RAE in the bottom figure) as the average error across all samples in the same bin. The green histogram in the background shows instead the distribution of the samples across the bins. (See more on this at the end of this section.)

The plots clearly show that, for CC, PCC, SVM(KLD), SVM(NKLD), SVM(Q), as well as for the newly added SVM(AE) and SVM(RAE), this error increases, in a very substantial manner as distribution shift increases. A common characteristic of this group of methods, that we will dub the “unadjusted” methods, is that none of them attempts to adjust the counts resulting from the classification of data items, thus resulting in quantification systems that behave reasonably well for test set class prevalence values close to the ones of the training set (i.e., for low values of distribution shift), but that tend to generate large errors for higher values of shift. The obvious conclusion is that failing to adjust makes the method not robust to high amounts of distribution shift, and that the reason why some unadjusted methods were successful in the evaluation of [GS2016] is that this latter confronted the methods with very low amounts of distribution shift. In fact, it is immediate to note from Figs 1 and 2 that, when distribution shift is between 0.020 and 0.1055 (the values of distribution shift that the experiments of [GS2016] tackled – the region of Figs 1 and 2 between the two vertical dotted lines encloses values of shift up to that level), the difference in performance between different quantification methods is small.

In our plots, by contrast, methods ACC, PACC, SLD, along with the newly added HDy, QuaNet, E(PACC)_AE_, and E(PACC)_Ptr_, form a second group of methods, that we will dub the “adjusted” methods, since they all implement, in one way or another, different strategies for post-processing the class prevalence estimations returned by base classifiers. The quantification error displayed by the “adjusted” methods remains fairly stable across the entire range of distribution shift values, which is clearly the reason of their success in the APP-based evaluation we have presented here.

**Fig 3:**
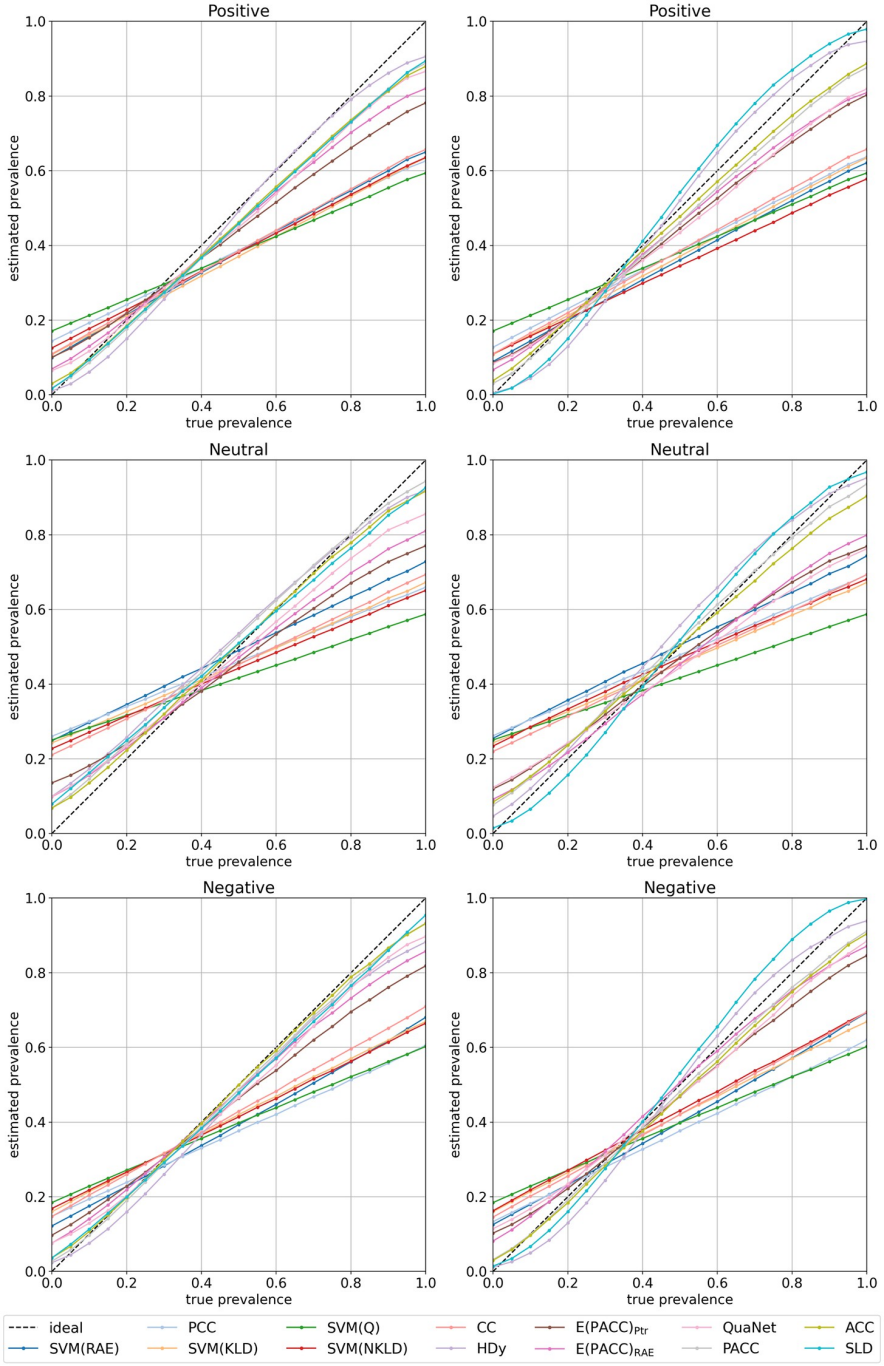
Estimated prevalence as a function of true prevalence according to various quantification methods. Results are displayed separately for classes ⊕ (top), ⊙ (middle), and ⊖ (bottom), with methods optimized for according to AE (left) and RAE (right).

Fig 3 shows the estimated class prevalence value (*y* axis) that each method delivers, on average across all test samples and all datasets, for each true prevalence (*x* axis); results are displayed separately for each of the three target classes and for methods optimized according to either AE or RAE. Note that the ideal quantifier (i.e., one that makes zero-error predictions) would be represented by the diagonal (0,0)-(1,1), here displayed as a dotted line. These plots support our observation that two groups of methods, the “adjusted” vs. the “unadjusted”, exist (this is especially evident for the ⊕ and the ⊖ classes, where they originate two quite distinct bundles of curves), and show how the unadjusted methods fail to produce good estimates for the entire range of prevalence values. As could be expected, all methods intersect approximately in the same point, which corresponds to the average training prevalence of the class across all datasets (*p*_*L*_(⊕) = 0.278, *p*_*L*_(⊙) = 0.426, *p*_*L*_(⊖) = 0.296), given that all methods tend to produce low error (hence similar values) for test class prevalence values close to the training ones.

**Fig 4:**
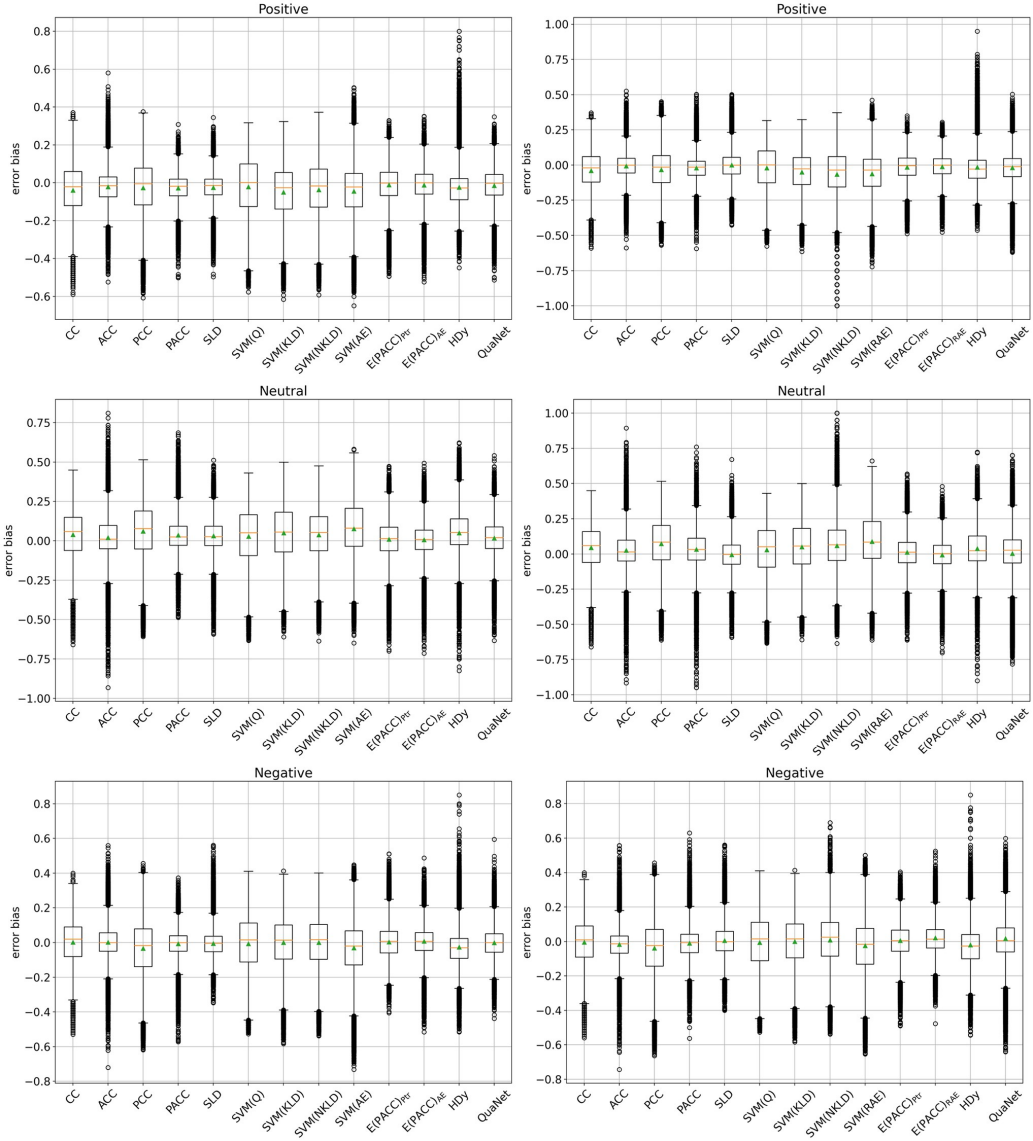
Box-plots of the error bias (signed error). Results are displayed separately for classes ⊕ (top), ⊙ (middle), and ⊖ (bottom), with methods optimized for according to AE (left) and RAE (right).

Fig 4 displays box-plot diagrams for the error bias (i.e., for the signed error between the estimated prevalence value and the true prevalence value) for all methods and independently for each class, as averaged across all datasets and test samples. The “adjusted” methods show lower error variance, as witnessed by the fact that their box-plots (indicating the first and third quartiles of the distribution) tend to be squashed and their whiskers (indicating the maximum and minimum, disregarding outliers) tend to be shorter. Some methods tend to produce many outliers (see, e.g., ACC and PACC in the ⊙ class), which might be due to the fact that the adjustments that those methods perform may become unstable in some cases. (This instability is well known in the literature, and has indeed motivated the appearance of dedicated methods that counter the numerical instability that some adjustments may produce in the binary case; see, e.g., [6, 39].) Overall, PACC and SLD, the two strongest methods among the quantification systems we have tested, seem to be also the methods displaying the smallest bias across the three classes.

As a final note, the reader might wonder why, for certain well-performing methods, quantification error even seems to *decrease* for particularly high values of distribution shift (see e.g., ACC, PACC, SLD in Fig 1 or SLD and ACC in Fig 2). The answer is that quantification error values for very high levels of shift are, in our experiments, not terribly reliable, because (as clearly shown by the green histograms in Figs 1 and 2) they are averages across *very few* data points. To see this, note that the values of AE range (see [27]) between 0 (best) and

$$\begin{array}{c} \begin{array}{cc} & \frac{2(1-{\text{min}}_{y\in Y}p\left(y\right))}{\left|Y\right|} \end{array} \end{array}$$ (10)

(worst), which in our ternary case means $\frac{2}{3}\left(1-0\right)=0.\bar{6}$ (because we indeed have test samples in which the prevalence of at least one class is 0). However, there are many more samples with extremely low AE values than samples with extremely high AE values; for instance, out of the 11 × 5,775 = 63,525 samples that we have generated in our experiments (see Section 2.3), there are only 25 whose value of distribution shift is comprised in the interval $\left[0.60,0.6\bar{6}\right]$, while there are no fewer than 3,300 whose value is comprised in the interval $\left[0.00,0.0\bar{6}\right]$, even if the two intervals have the same width. To see why, note for instance that we can reach an AE value of $0.\bar{6}$ only when one of the classes in the training set has a prevalence value of 0 (see Eq 10), while an AE value of 0 can be reached for all training sets. As a result, the average AE values at the extreme right of the plots in Figs 1 and 2 (say, those beyond *x* = 0.55) are averages across very few data points, and are thus unstable and unreliable. This does not invalidate our general observations, though, since each quantification method we test displays, on the [0.00,0.55] interval, a very clear, unmistakable behaviour.

### 4.1 Difference between systems and their statistical significance

Concerning the differences between rank positions in the experimentations of this paper and of [GS2016] reported in Tables 4 and 5, we want to remark that they are just meant to provide an additional, quick reading of how differently the methods perform in the two experimentations, and should not be considered a substitute of the original numerical results from which they are obtained, as available from Tables 2 and 3.

While those differences are only qualitative in nature, we also want to investigate differences between systems from a quantitative way. We thus study, separately in our batch of experiments and in the experiments of [GS2016], the extent to which the differences in performance amongst methods (as quantified by differences in error scores, and *not as differences of rankings*) are indeed significant (in a statistical sense) depending on the evaluation protocol. The results of the pairwise comparisons (in terms of a two-sided Wilcoxon signed-rank test on related paired samples) are reported in Tables 6 and 7, for AE and RAE, respectively.

**Table 6:** Pairwise comparisons, according to the Wilcoxon test, for the experiments run in this work (left) and the experiments from [GS2016] (right) when adopting AE as the evaluation measure. The symbol ‘>’ (resp. ‘<’) indicates that the method in the row is better than (resp., is worse than) the method in the column, with a confidence level of 99%, while symbol ‘≈’ indicates instead that the difference between the two is not significant. Symbols ‘≫’ and ‘≪’ are used in place of ‘>’ and ‘<’ when the differences in performance are found to be significant at a higher confidence level of 99.9%.

	CC	ACC	PCC	PACC	SLD	SVM(Q)	SVM(KLD)	SVM(NKLD)		CC	ACC	PCC	PACC	SLD	SVM(Q)	SVM(KLD)	SVM(NKLD)
CC	-	≪	≫	≪	≪	≫	≫	≫	CC	-	≈	≪	≈	≈	≈	≈	≈
ACC	≫	-	≫	≪	≪	≫	≫	≫	ACC	≈	-	≈	≈	≈	>	≈	>
PCC	≪	≪	-	≪	≪	≫	≪	≪	PCC	≫	≈	-	≈	>	≫	≈	>
PACC	≈	≈	≈	-	≈	≫	≈	>	PACC	≈	≈	≈	-	≈	>	≈	≈
SLD	≫	≫	≫	≪	-	≫	≫	≫	SLD	≈	≈	<	≈	-	≈	≈	≈
SVM(Q)	≫	≫	≫	≪	≪	-	<	≈	SVM(Q)	≈	<	≪	<	≈	-	≈	≈
SVM(KLD)	≪	≪	≫	≪	≪	≫	-	≪	SVM(KLD)	≈	≈	≈	≈	≈	≈	-	≈
SVM(NKLD)	≪	≪	≫	≪	≪	≫	≫	-	SVM(NKLD)	≈	<	<	≈	≈	≈	≈	-

**Table 7:** Pairwise comparisons, according to the Wilcoxon test, for the experiments run in this work (left) and the experiments from [GS2016] (right) when adopting RAE as the evaluation measure. The symbol ‘>’ (resp. ‘<’) indicates that the method in the row is better than (resp. is worse than) the method in the column, with a confidence level of 99%, while symbol ‘≈’ indicates instead that the difference between the two is not significant. Symbols ‘≫’ and ‘≪’ are used in place of ‘>’ and ‘<’ when the differences in performance are found to be significant at a higher confidence level of 99.9%.

	CC	ACC	PCC	PACC	SLD	SVM(Q)	SVM(KLD)	SVM(NKLD)		CC	ACC	PCC	PACC	SLD	SVM(Q)	SVM(KLD)	SVM(NKLD)
CC	-	≪	≫	≪	≪	≫	≫	≫	CC	-	≈	≈	≈	>	>	≈	≈
ACC	≫	-	≫	≈	≪	≫	≫	≫	ACC	≈	-	≈	≈	≈	≫	≈	≫
PCC	≪	≪	-	≪	≪	≫	≪	≪	PCC	≈	≈	-	≈	>	>	≈	>
PACC	≫	≈	≫	-	≪	≫	≫	≫	PACC	≈	≈	≈	-	≈	≫	≈	>
SLD	≫	≫	≫	≫	-	≫	≫	≫	SLD	<	≈	<	≈	-	≈	≈	≈
SVM(Q)	≪	≪	≪	≪	≪	-	≪	≪	SVM(Q)	<	≪	<	≪	≈	-	<	≈
SVM(KLD)	≪	≪	≫	≪	≪	≫	-	≫	SVM(KLD)	≈	≈	≈	≈	≈	>	-	>
SVM(NKLD)	≪	≪	≫	≪	≪	≫	≪	-	SVM(NKLD)	≈	≪	<	<	≈	≈	<	-

Something that jumps to the eye is that the results derived from our experimentation tend to be much more conclusive (in the sense of statistical significance) when it comes to judging the superiority of one method over another. Indeed, all differences resulting from our experiments, as reported in Table 6, turn out to be statistically significant at a very high level of confidence, while no fewer than 75% of the comparisons obtainable from the results in [GS2016] are inconclusive; in Table 7), instead, only 2 differences out of 56 turn out to be not significant in our experiments (namely, the comparisons between PACC and ACC), while this happens in 34 cases out of 56 for the experiments of [GS2016]. After all, it is not surprising that a test of statistical significance deems more significant the differences found for a set of experiments based on 63,525 samples than for a set of experiments based on 11 samples.

## 5 Conclusions

A re-evaluation of the relative merits of different quantification methods on the tweet sentiment quantification task was necessary, due to the insufficient number of test samples which [GS2016] used. We have shown that the experimentation previously conducted in [GS2016] was weak, since the authors of [GS2016] overlooked the fact that the experimental protocol they followed led them to conduct their evaluation on a radically insufficient amount of test *samples*. We have then conducted a re-evaluation of the same methods on the same datasets according to a robust and now widely accepted experimental protocol, which has lead to an experimentation on a number of test samples 5,775 times larger than the one of [GS2016]. In addition to these experiments, we have also tested some further methods, some of which had appeared after [GS2016] was published. This experimentation was also necessary because some evaluation functions (such as KLD and NKLD) that had been used in [GS2016] are now known to be unsatisfactory, and their use should thus be deprecated in favour of functions such as AE and RAE.

Due to the presence, in the test data, of samples characterised by class prevalence values very different from those of the training set, the results of our re-evaluation have radically disconfirmed the conclusions originally drawn by the authors of [GS2016], showing that the methods (e.g., PCC) who had emerged as the best performers in [GS2016] tend to behave well only in situations characterised by very low distribution shift. (The test samples used in [GS2016] were indeed all of this type.) On the contrary, when distribution shift increases, other methods (such as SLD) are to be preferred. In particular, our experiments do justice to the SLD method, which had obtained fairly bland results in the experiments of [GS2016], and which now emerges as the true leader of the pack, thanks to consistently good performance across the entire spectrum of distribution shift values.

## Supporting information

S1 Appendix (ZIP)
