# Supplementary material for: Tweet sentiment quantification: An experimental re-evaluation
Source: PLoS One. 2022 Sep 16;17(9):e0263449. doi: 10.1371/journal.pone.0263449 (PMC9481048; doi:10.1371/journal.pone.0263449)
Supplement: S1 Appendix — (ZIP) [file pone.0263449.s001.zip › Appendix.pdf]

## A Tables of results obtained in [GS2016]

| Dataset | CC | ACC | PCC | PACC | SLD | SVM(Q) | SVM(KLD) | SVM(NKLD) |
|---|---|---|---|---|---|---|---|---|
| GASP | 0.019 | 0.015 | 0.010 | **0.009** | 0.023 | 0.064 | 0.017 | 0.050 |
| HCR | 0.052 | 0.024 | **0.005** | 0.033 | 0.089 | 0.127 | 0.041 | 0.060 |
| OMD | 0.052 | 0.024 | **0.005** | 0.010 | 0.065 | 0.062 | 0.030 | 0.044 |
| Sanders | 0.067 | 0.034 | 0.015 | 0.030 | 0.071 | 0.110 | **0.013** | 0.095 |
| SemEval2013 | 0.100 | 0.081 | **0.034** | 0.081 | 0.119 | 0.078 | 0.072 | 0.071 |
| SemEval2014 | 0.104 | 0.058 | 0.100 | **0.053** | 0.081 | 0.102 | 0.084 | 0.084 |
| SemEval2015 | 0.110 | 0.106 | **0.046** | 0.101 | 0.120 | 0.126 | 0.118 | 0.116 |
| SemEval2016 | 0.050 | 0.054 | **0.038** | 0.086 | 0.065 | 0.120 | **0.038** | 0.083 |
| SST | 0.033 | 0.049 | **0.028** | 0.084 | 0.037 | 0.067 | 0.041 | 0.075 |
| WA | 0.043 | 0.041 | 0.034 | **0.028** | 0.039 | 0.080 | 0.065 | 0.039 |
| WB | 0.013 | 0.023 | **0.012** | 0.017 | 0.024 | 0.025 | 0.061 | 0.053 |
| Average | 0.059 | 0.046 | **0.030** | 0.048 | 0.067 | 0.087 | 0.053 | 0.070 |

**Table 1.** Results from [GS2016] in terms of AE. **Boldface** indicates the best result obtained on the dataset.

| Dataset | CC | ACC | PCC | PACC | SLD | SVM(Q) | SVM(KLD) | SVM(NKLD) |
|---|---|---|---|---|---|---|---|---|
| GASP | 0.130 | 0.104 | 0.068 | 0.060 | 0.159 | 0.440 | **0.053** | 0.342 |
| HCR | 0.182 | 0.103 | **0.020** | 0.144 | 0.309 | 0.460 | 0.219 | 0.232 |
| OMD | 0.153 | 0.075 | **0.009** | 0.029 | 0.189 | 0.183 | 0.100 | 0.128 |
| Sanders | 0.268 | 0.131 | **0.060** | 0.117 | 0.285 | 0.436 | 0.063 | 0.397 |
| SemEval2013 | 0.309 | 0.248 | **0.151** | 0.263 | 0.392 | 0.278 | 0.172 | 0.276 |
| SemEval2014 | 0.321 | 0.236 | 0.428 | 0.257 | 0.352 | 0.368 | **0.227** | 0.337 |
| SemEval2015 | 0.288 | 0.297 | **0.197** | 0.273 | 0.295 | 0.476 | 0.379 | 0.472 |
| SemEval2016 | 0.177 | 0.225 | 0.155 | 0.350 | 0.213 | 0.516 | **0.151** | 0.325 |
| SST | 0.124 | 0.169 | **0.107** | 0.230 | 0.119 | 0.234 | 0.146 | 0.250 |
| WA | 0.127 | 0.120 | 0.099 | **0.082** | 0.115 | 0.233 | 0.196 | 0.136 |
| WB | 0.040 | 0.072 | **0.039** | 0.051 | 0.077 | 0.077 | 0.179 | 0.176 |
| Average | 0.193 | 0.162 | **0.121** | 0.169 | 0.228 | 0.336 | 0.171 | 0.279 |

**Table 2.** Results from [GS2016] in terms of RAE. **Boldface** indicates the best result obtained on the dataset.
